# Research on a Visual Electronic Nose System Based on Spatial Heterodyne Spectrometer

**DOI:** 10.3390/s18041188

**Published:** 2018-04-13

**Authors:** Wenli Zhang, Fengchun Tian, An Song, Youwen Hu

**Affiliations:** College of Communication Engineering, Chongqing University, 174 Sha Pingba, Chongqing 400044, China; 20151201003@cqu.edu.cn (W.Z.); 20161202038t@cqu.edu.cn (A.S.); hyw_cqu@163.com (Y.H.)

**Keywords:** visual e-nose, SHS, gas sensing, feature extraction, pattern recognition

## Abstract

Light absorption gas sensing technology has the characteristics of massive parallelism, cross-sensitivity and extensive responsiveness, which make it suitable for the sensing task of an electronic nose (e-nose). With the performance of hyperspectral resolution, spatial heterodyne spectrometer (SHS) can present absorption spectra of the gas in the form of a two dimensional (2D) interferogram which facilitates the analysis of gases with mature image processing techniques. Therefore, a visual e-nose system based on SHS was proposed. Firstly, a theoretical model of the visual e-nose system was constructed and its visual maps were obtained by an experiment. Then the local binary pattern (LBP) and Gray-Level Co-occurrence Matrix (GLCM) were used for feature extraction. Finally, classification algorithms based on distance similarity (Correlation coefficient (CC); Euclidean distance to centroids (EDC)) were chosen to carry on pattern recognition analysis to verify the feasibility of the visual e-nose system.

## 1. Introduction

As a representative of artificial olfactory technology, e-nose can provide an objective assessment of smell which is widely used in food safety [1,2,3,4,5], disease diagnosis [6,7,8,9,10], environmental monitoring [11,12,13,14,15,16], public safety [17,18], etc. Artificial olfactory technology [19,20] has made great achievements in the past two decades, but compared with human olfactory system, there is still a gap, mainly due to the imperfections of the e-nose system, such as small number of sensing units, narrow response range and so on [21,22,23]. Light absorption gas sensing technology [24,25] has the characteristics of massive parallelism, cross-sensitivity, extensive and fast responsiveness, which make it suitable for the sensing task of an e-nose. So, if light absorption gas sensing technology is applied to e-nose system, the problems of fewer units, long response time, short life, poor repeatability and harsh environmental requirements of typical e-nose (such as PEN3, Alpha MOS, etc.) will be solved.

At present, almost all light absorption gas detection systems [26,27,28,29,30] use a grating spectrometer to detect gases [31,32,33], but the system has a problem that must be considered: there are constraints between the spectral range and the resolution of spectrometer. With the performance of hyperspectral resolution, lack of moving parts, low requirements for components and high etendue, spatial heterodyne spectrometer (SHS) [34,35] is widely used in trace gasses detection [36], astrophysical observation [37]. However, the output of the SHS is a 2D interferogram, and the target spectral information is implicit in the interferogram. According to the traditional data processing method, the interferogram needs to be baseline removed, apodized, phase corrected and inverse Fourier transformed to reconstruct the input spectrum [38,39]. According to the basic principle of e-nose [19,20], the gas’s response can be replaced by its corresponding feature instead of raw data. Therefore, under the premise of a one-to-one correspondence between the interferogram and the target spectrum, using the characteristics of the interferogram to replace the original gas information as the sensor response of the e-nose can not only apply the mature image processing technology to the data processing of the e-nose, but also reduce the complexity of data processing and improve the efficiency of the e-nose.

An innovative visual e-nose system based on SHS was proposed. Firstly, a mathematical model of a visual gas sensing mechanism was built and the feasibility of the model appropriate to the sensing task of e-nose was demonstrated. Secondly, one dimensional (1D) spectral data of different test gases were obtained by light absorption experiment and their 2D response interferograms were obtained by simulation. Thirdly, image feature extraction algorithms, principal component analysis (PCA) and classifiers were used for processing of the interferogram.

## 2. Visual Gas Sensing Mechanism Based on SHS

### 2.1. Wide Spectral Spatial Heterodyne Spectrometer

#### 2.1.1. Wide Spectral Spatial Heterodyne Spectrometer

**Figure 1:**
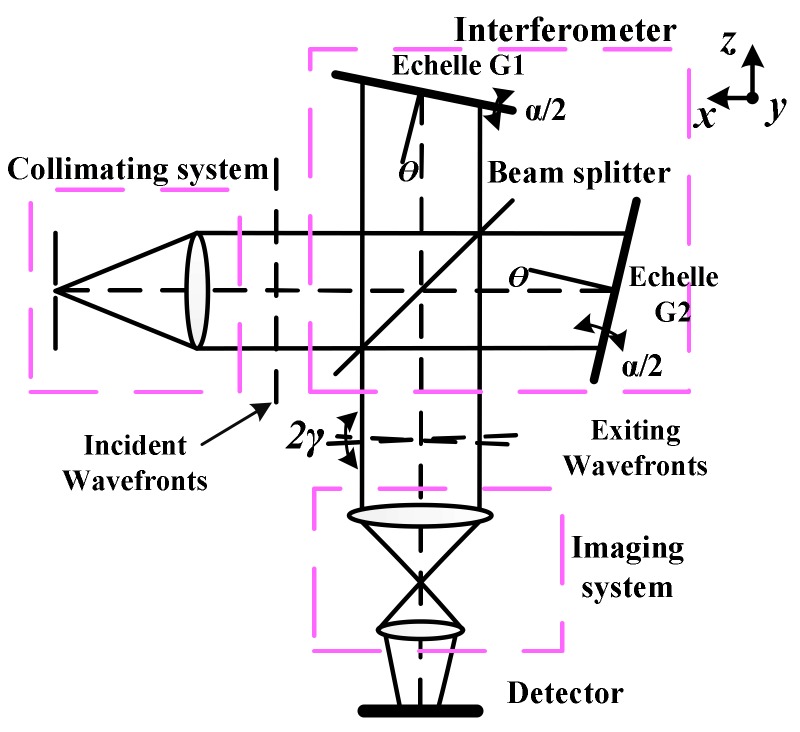
Structure diagram of 2D spatial heterodyne spectrometer (SHS).

The structure of wide spectral SHS (WS-SHS) is shown in Figure 1. A WS-SHS is essentially a Michelson interferometer with the mirrors replaced by echelle gratings that are fixed at the zero-path difference position for a series wave-numbers $\left(\sigma_{0m}\right)$ at the Littrow angle (θ) of the echelle gratings (see Figure 1). The recombined wave-fronts at $\sigma_{0m}$ are parallel; however, other wave-numbers are dispersed by the echelle gratings and rotated in opposite directions by an angle γ. In order to remove the ambiguity of multi-dispersion orders in recovered spectra, the echelle gratings are tilted perpendicular to the dispersion plane with the angle α/2. The interferential fringes become two dimensional patterns. Using the small-angle approximation, the ideal WS-SHS interferogram can be written as [34,37]:

(1) $$I(x,y)=\sum_{m}\int_{0}^{\infty }B(\sigma )F_{m}(\sigma )(1+\cos(2\pi (4(\sigma -\sigma_{0m})\tan\theta \cdot x+\alpha \sigma \cdot y)))d\sigma ,$$

where B(σ) is the wavenumber-dependent spectral radiances, $\sigma_{0m}$ is the Littrow wavenumber for order m which corresponds to the grating angle θ, x is the pixel location of detector along dispersion direction, y is the pixel location of detector perpendicular to dispersion direction, and $F_{m}(\sigma )$ is the diffractive efficiency of grating for order m. The fringes recorded for arbitrary wavenumber are rotated with respect to the x axis by an angle η where $\tan\eta =-f_{x}/f_{y}$ $\left(f_{x}=4(\sigma -\sigma_{0})\tan\theta ,f_{y}=\alpha \sigma \right)$.

#### 2.1.2. Basic Properties of the WS-SHS System

(1) Spectral resolution

The spectral resolution for WS-SHS depends on the optical path difference between two interfering beams. As shown in Figure 1, the maximum optical path difference along axis x is $U_{x\max}=2W\sin\theta$. Then the theoretical spectral resolution of WS-SHS is [37]:

(2) $$\delta \sigma =1/2U_{x\max}=1/4W\sin\theta ,$$

where W is the effective width of the grating perpendicular to the dispersion direction.

(2) Spectral range

At the same time, the spectral range for every order diffraction of the gratings is $2\sigma_{01}$, and the lower limit of the numbers for pixel of detectors along the direction of x is [37]:

(3) $$N_{x}\geq 2\sigma_{01}/\delta \sigma .$$

In the same way, the resolution of the transform in y must be high enough to distinguish adjacent orders from one another by a differential of at least one fringe. If the maximum diffraction order is $m_{\max}$, then a lower limit of the numbers for pixel of detectors along the direction of y is [37]:

(4) $$N_{y}\geq 4m_{\max}.$$

Equations (3) and (4) limit the minimum number of samples in each direction. At this point, the spectral range of the WS-SHS is:

(5) $$\Delta \sigma =\sum_{m}\left(\sigma_{0m}\pm \sigma_{01}\right).$$

When m is from one to hundreds, the spectrum detection range of WS-SHS will be from ultraviolet to infrared.

### 2.2. Sensing Mechanism of the Visual E-Nose

The visual gas sensing mechanism based on SHS combines the basic principles of spectroscopy and SHS. One-dimensional characteristic spectra of the test gas are obtained by light absorption gas detection system. Then, the characteristic spectra are presented in the form of 2D interferogram by SHS. The mathematical model is as follows:

Absorption spectrum of the test gas are obtained from Lambert–Beer law [40],

(6) $$B_{out}(\sigma )=B_{in}(\sigma )e^{-\alpha (\sigma )CL},$$

where $B_{in}(\sigma ),\;B_{out}(\sigma )$ represent input spectrum and absorption spectrum of the light absorption system respectively. If they are input into the SHS system, and $f_{x}=4(\sigma -\sigma_{0})\tan\theta ,\;f_{y}=\alpha \sigma$, the output of SHS is obtained as follows:

(7) $$I_{in}(x,y)=\sum_{m}\int_{0}^{\infty }B_{in}(\sigma )F_{m}(\sigma )(1+\cos(2\pi (f_{x}\cdot x+f_{y}\cdot y)))d\sigma ,$$

(8) $$I_{out}(x,y)=\sum_{m}\int_{0}^{\infty }B_{out}(\sigma )F_{m}(\sigma )(1+\cos(2\pi (f_{x}\cdot x+f_{y}\cdot y)))d\sigma .$$

Taking Equation (6) into Equation (8), the response of absorption spectrum is obtained as follows:

(9) $$I_{out}(x,y)=\sum_{m}\int_{0}^{\infty }B_{in}(\sigma )e^{-\alpha (\sigma )CL}F_{m}(\sigma )(1+\cos(2\pi (f_{x}\cdot x+f_{y}\cdot y)))d\sigma .$$

Comparing Equations (7) and (9), it is found that the input interferogram of the system is the Fourier transform of the input spectrum $B_{in}(\sigma )$ and the absorption interferogram is the Fourier transform of absorption spectrum $B_{out}(\sigma )$. Set $G(\sigma ,x,y)=B_{in}(\sigma )F_{m}(\sigma )(1+\cos(2\pi (f_{x}\cdot x+f_{y}\cdot y)))$, and define a transmittance map (T-map) as:

(10) $$T(x,y)=\frac{I_{out}(x,y)}{I_{in}(x,y)}=\frac{\sum_{m}\int_{0}^{\infty }G(\sigma ,x,y)e^{-\alpha (\sigma )CL}d\sigma }{\sum_{m}\int_{0}^{\infty }G(\sigma ,x,y)d\sigma },$$

where T(x,y) is a function of pixel position which reflects characteristic information of the test gas. Meanwhile, from the definition of SHS, it can be found that I(x,y) is formed by the superposition of interference fringes of different diffraction orders, and fringe structure and fringe period depend on the spectral distribution of the test gas. For Equation (10), if the system is determined, L is a constant. Then for different kinds of gases, α(σ) is different and assume that C is a constant, the difference of T(x,y) is reflected in fringe structure, fringe period of the T-map which can be used as the basis of gas type judgment. For the same kind of gas, α(σ) is same, so the fringe structure and fringe period is unchanged. When C is different, there is a difference of T(x,y) in brightness, which can be the basis of gas concentration judgment. Therefore, it is feasible in theory to use T(x,y) as a sensing mechanism for constructing an e-nose system. In such condition, each pixel of T(x,y) plays a role of virtual sensor, then the huge number of pixels obviously increases the number of sensing units in the system.

## 3. Visual E-Nose System Based on SHS

A theoretical model of the visual e-nose system was established in Section 2. In order to verify the effectiveness of the system, T-maps are obtained through experiments and objective parameters are selected to evaluate the system performance in this section.

### 3.1. Construction of Visual E-Nose System

#### 3.1.1. System Structure

**Figure 2:**
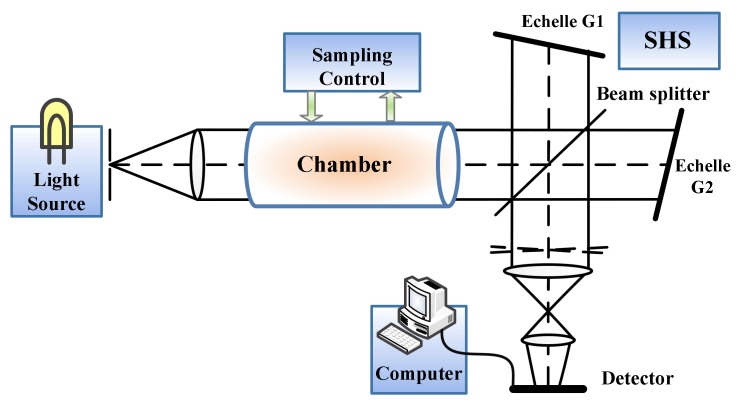
Structure of the visual e-nose system.

Structure of the visual e-nose system is shown in Figure 2.

Light source is the sensing medium of the system providing the energy and characteristic spectral lines required by the system. Chamber is the place where light source and gases react with each other. Sampling control module controls the type and concentration of test gas. SHS is used to generate and collect 2D interferogram. Computer realizes the analysis of interferograms.

#### 3.1.2. Flowchart of the Visual E-Nose

**Figure 3:**
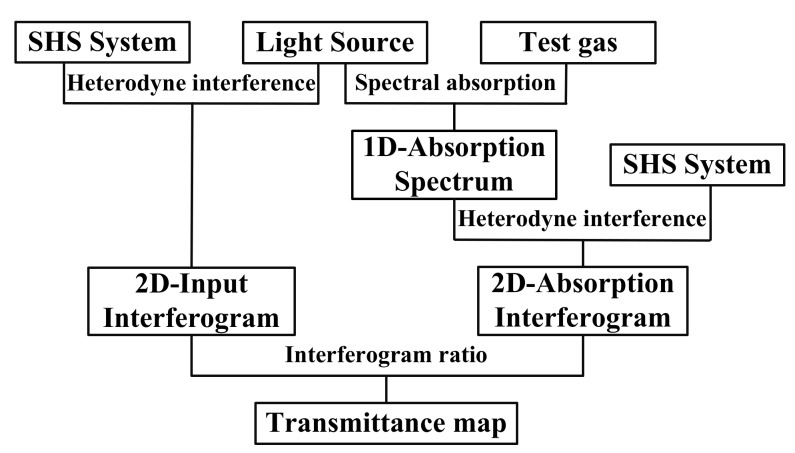
Flowchart of the visual e-nose system.

Flowchart of the visual e-nose system is shown in Figure 3.

Firstly, a light source was input into the SHS system to get its 2D input interferogram. Then, the light source was input into light absorption gas detection system to get 1D absorption spectrum and it was input into the SHS system to get the 2D absorption interferogram. Finally, the T-map was obtained by taking the input interferogram and the absorption interferogram into Equation (10).

### 3.2. Experiment

From the basic principle of visual e-nose, we can see that with the increase of the diffraction order of Echelle gratings, the system can be used not only in UV–visible but also in infrared band, while in this article, UV–visible band is selected to verify the feasibility and effectiveness of the system. In addition, NO_2_, SO_2_, mixture of NO_2_ and SO_2_ (NO_2_ + SO_2_), C_6_H_6_, C_7_H_8_ were selected taking into account the effective working range of the spectrum and the gas performance in this band.

#### 3.2.1. Experimental Steps

**Figure 4:**
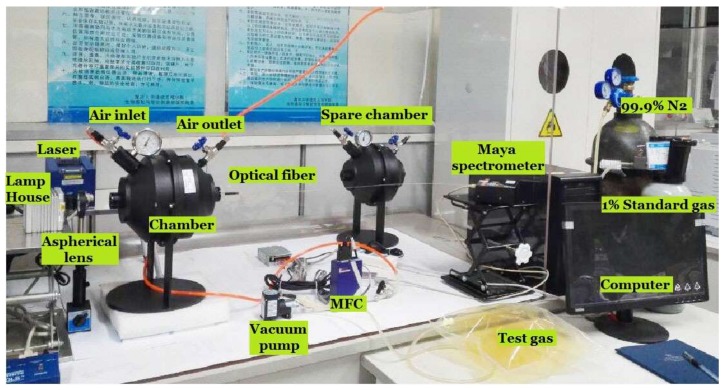
Experiment platform of light absorption gas detection system.

The experimental platform of light absorption gas detection system is shown in Figure 4, and the technical parameters of the system are as shown in Table 1.

The technical parameters in Table 1 show that the light absorption gas detection system is more suitable to study the gas that has absorption in the spectral range of 200–1100 nm, and the detection range of the system is mainly limited by the detection range of the spectrometer. From Section 2.1, it is noticed that the proposed visual e-nose system can be also applied in other wavelength ranges as long as the device is coincident.

The detailed experimental processes were as follows:

(1) Obtained 1D spectrum

Step 1: When the light source (EQ-99X, Energetiq, Woburn, MA, USA, spectral range is 190–2100 nm) is off, use the grating spectrometer (Maya 2000Pro, Ocean Optics, Largo, FL, USA, spectral range is 200–1100 nm, resolution is 0.54 nm) to record the background spectrum. Then turn on the light source and record the input spectrum.

Step 2: Fill the chamber with the test gases (NO_2_, SO_2_, NO_2_ + SO_2_, C_6_H_6_, C_7_H_8_) by Vacuum pump and MFC (The concentrations of NO_2_ are 0.2‰, 0.4‰, 0.6‰ respectively; The concentrations of SO_2_ are 0.3‰, 0.6‰, 0.9‰ respectively; The concentrations of NO_2_ + SO_2_ are 3.2‰ + 0.1‰, 3.5‰ + 0.3‰, 3.0‰ + 0.6‰ respectively; The concentrations of C_6_H_6_ and C_7_H_8_ are all 3‰). In addition, the 1D background spectrum, input spectrum and absorption spectrum were collected, respectively.

**Figure 5:**
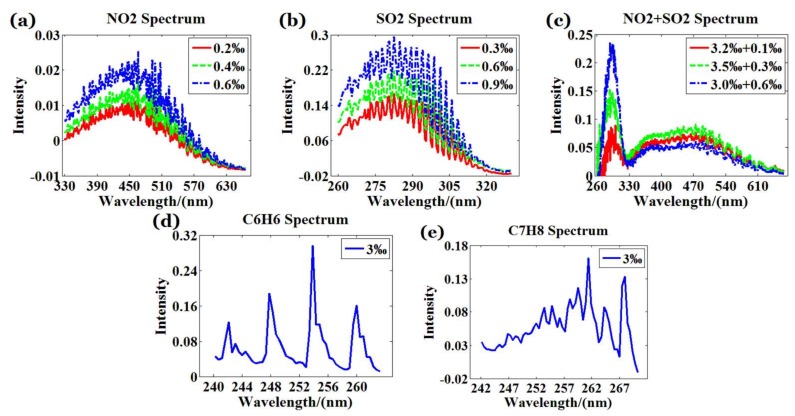
One-dimensional characteristic curve of test gases (**a**) NO_2_ (**b**) SO_2_ (**c**) NO_2_ + SO_2_ (**d**) C_6_H_6_ (**e**) C_7_H_8_.

Step 3: Background interference was removed by subtracting the background spectrum from the input spectrum and the absorption spectrum. Then, take the processed input spectrum and absorption spectrum into Equation (6) to obtain the characteristic curve of the corresponding gas as shown in Figure 5.

Step 4: According to the characteristic spectral range of the test gases provided in the HITRAN standard database [41], 240–650 nm is chosen as the effective range of the WS-SHS system input spectrum.

(2) Obtained 2D T-maps

Step 5: Assume that the blazed angle of the Echelle grating is 63°, the groove density is 31.6l/mm, the effective width is 20 mm, and the input spectral range is 240–650 nm. Then the resolution of WS-SHS calculated by Section 2.1.2 is 0.014 mm^−1^, the minimum detector’s pixel size is 600 × 1400, the maximum diffraction order is 150. However, the spectral resolution in the interferogram is 0.54 nm because of the limitations brought by the simulation experiment.

Step 6: Take the processed input spectrum input into WS-SHS to get 2D input interferogram. The interferogram size is 600 × 1400, which means that the number of sensors for this visual e-nose system is 600 × 1400.

**Figure 6:**
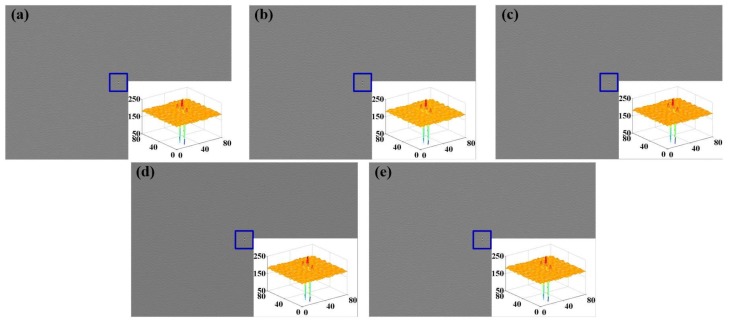
Interferograms of test gases (**a**) NO_2_ (**b**) SO_2_ (**c**) NO_2_ + SO_2_ (**d**) C_6_H_6_ (**e**) C_7_H_8_.

Step 7: Take the processed absorption spectrum of the test gases with different types and concentrations into WS-SHS to obtain 2D absorption interferograms. For each kind of gas, 32 sets of data were collected and only the first one was shown in Figure 6.

Observing Figure 6, the contrast of the interferogram is low, and the main reasons for this is that [32] the interferogram of WS-SHS is formed by the superposition of interferograms of different diffraction orders. The lager the number of the superposition is, the lower the contrast of the interferogram is. In this system, the input spectrum (240–650 nm) of WS-SHS covers the UV–visible band at the same time, and the diffraction order of the Echelle grating is 150. That is, the above interferogram is composed of 150 interferograms with different diffraction orders, which seriously reduce the contrast of the interferogram.

#### 3.2.2. Acquisition and Analysis of Sensing Data

**Figure 7:**
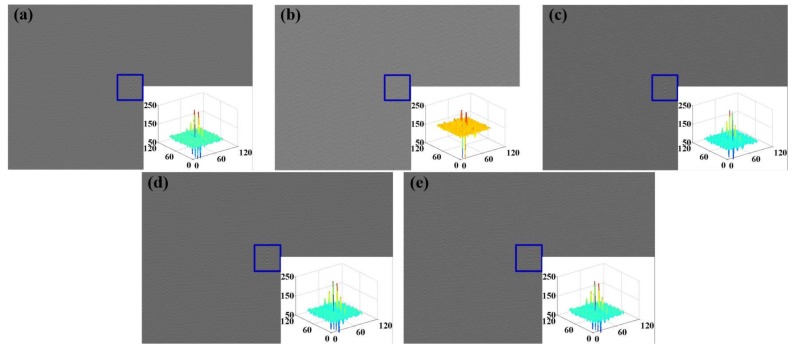
T-maps of test gases (**a**) NO_2_ (**b**) SO_2_ (**c**) NO_2_ + SO_2_ (**d**) C_6_H_6_ (**e**) C_7_H_8_.

The interferograms of input spectrum and absorption spectrum of different gases at different concentrations were acquired by Section 3.2.1. Then the T-maps could be acquired by bringing the above data into Equation (10) (shown in Figure 7).

According to the T-maps shown in Figure 7, it was found that different spectra have different T-maps. The difference is reflected in interference fringe structure, fringe period. However, because of the wide spectral range of the input light, the fringe contrast of the T-map is low which affects the subjective observation. To acquire objective analysis of the results, correlation coefficient [42] is chosen to evaluate the sensing data of the visual e-nose. Results of the sensing data using correlation coefficient are shown in Table 2.

Comparing the correlation coefficient parameters in Table 2, it can be found that there are obvious differences among the sensing data of different gases, and that even the difference between the mixed gas and the single gas is large with correlation coefficients less than 1, which preliminarily verifies the feasibility of the visual e-nose system.

## 4. Analysis of the Visual E-Nose Sensing Data

### 4.1. Feature Extraction of Experiment Sensing Data

#### 4.1.1. Feature Extraction

Based on the theory of visual e-nose, the sensing data acquired by the system are images which are formed by the superposition of interferograms of different diffraction orders. Usually the scale of the image is very large, so the complexity of the system will be increased if the image is used directly as the input of pattern recognition. However, if characteristics of T-maps are extracted in the form of 1D vector and used as input of pattern recognition, the processing complexity of data will be reduced and the efficiency of the e-nose is going to be improved. At present, typical image feature extraction algorithms include local binary pattern (LBP), GLCM etc.

(1) Local binary pattern (LBP)

**Figure 8:**
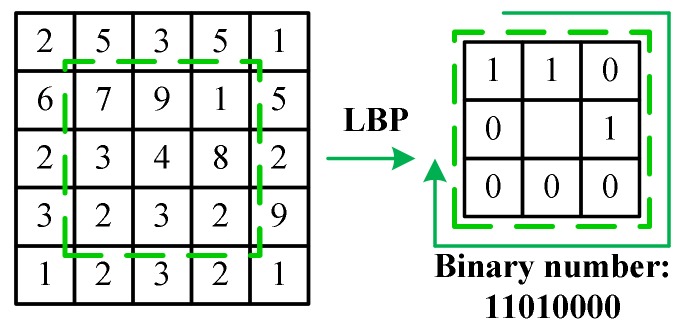
Example of obtaining the local binary pattern (LBP) micropattern for the region in the dotted box.

LBP [43] is defined as a grayscale invariant texture measure and is a useful tool to model texture images. The original LBP operator labels the pixels of an image by thresholding the three by three neighborhood of each pixel with the value of the central pixel and concatenating the results binomially to form a number. Figure 8 shows an example of obtaining an LBP micropattern when the threshold is set to zero. The histograms of these micropatterns contain information of the distribution of the edges, spots, and other local features in an image.

(2) Gray-Level Co-occurrence Matrix (GLCM)

GLCM is the statistical method of examining the textures that considers the spatial relationship of the pixels. The GLCM functions characterize the texture if an image by calculating how often pairs of pixel with specific values and in a specified spatial relationship occur in an image, creating a GLCM, and then extracting statistical measures from this matrix [44]. The common statistical parameters include angular second moment (ASM), contrast (CON), inverse difference moment (IDM), Entropy (ENT), Correlation (COR), Variance (VAR), Sum Average (SA) and Sum Entropy (SE) [45].

**Figure 9:**
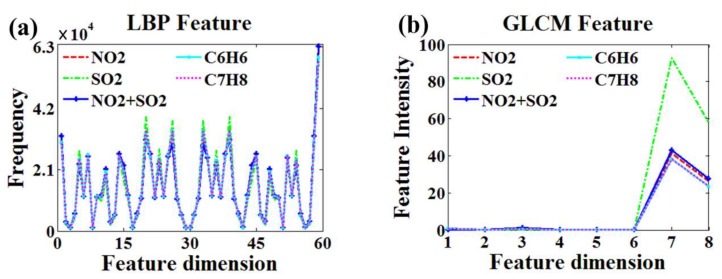
Results of different feature extraction algorithms. GLCM: Gray-Level Co-occurrence Matrix. (**a**) Feature extraction based on LBP (**b**) Feature extraction based on GLCM.

The features of T-maps extracted by LBP and GLCM are shown in Figure 9.

In Figure 9a, the feature dimension of LBP is 59, which represent 59 local binary patterns of T-maps and the vertical axis represents the number of occurrence of features. In Figure 9b, the feature dimension of GLCM is 8, which respectively represent ASM, CON, IDM, ENT, COR, VAR, SA and SE, the vertical axis indicates the amplitude of the feature. According to the analysis of Figure 9, compared with GLCM features, the data extracted by LBP have higher dimension, but its features are too similar, and the curves in the figure almost coincide.

#### 4.1.2. PCA Analysis

**Figure 10:**
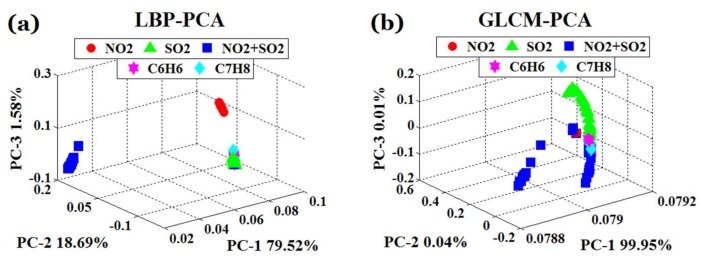
Principal component analysis (PCA) scattergram of different feature extraction algorithms. (**a**) PCA scatter diagram of the feature extracted based on LBP, (**b**) PCA scatter diagram of the feature extracted based on GLCM.

PCA [46] is a multivariate statistical technique that transforms multiple variables into a few principal components by dimensionality reduction. The principal components after PCA analysis can reflect most of the information of the original variables. In practical application, the researcher can select corresponding principal component by the cumulative contribution rate for subsequent data processing. In this section, PCA was used to analyze the features extracted by LBP and GLCM (shown in Figure 10).

In Figure 10, PC1, PC2, PC3 respectively represent the percentages of the original information contained in each principal component of PCA analysis, while the axes reflect the distribution of scattered points. From the first three PCA results (cf. Figure 10) we can see, in the PCA scattergram of LBP, that NO_2_ and NO_2_ + SO_2_ can be separated obviously, but the distribution of characteristic data of SO_2_, C_6_H_6_ and C_7_H_8_ overlaps seriously. In the PCA scattergram of GLCM, NO_2_ and SO_2_ can be separated obviously, the distribution of NO_2_ and NO_2_ + SO_2_ overlaps slightly, the discrimination of SO_2_, C_6_H_6_ and C_7_H_8_ is a little better than that of LBP, but the overlap still exists.

### 4.2. Type Recognition of the Experiment Sensing Data

#### 4.2.1. Classifiers and Experimental Data

(1) Classifiers

Correlation coefficient (CC) [47] and Euclidean distance to centroids (EDC) [48] were chosen to determine the gas type.

(2) Experimental data

In Section 3.2, the sensing data of test gases with different types and concentrations are obtained by experiment. In this section, Dataset is selected from the 2D T-maps (shown in Figure 7) for pattern recognition analysis. The specific dataset is shown in Table 3.

#### 4.2.2. Recognition and Analysis of Gas Type

Feature extraction and PCA dimension reduction were performed and the first three principal components of the feature vector (of which the accumulated contribution > 99%) were selected to construct the new dataset. Then Kennard–Stone sequential (KSS) algorithm [49] was used to allocate the new sample set to the training set and the test set according to the proportion of 7:3 and classification analysis was performed at last.

The classification accuracy of pattern recognition of Dataset using the CC and EDC algorithms is shown in Table 4.

Comparing the results of Dataset in Table 4, the following conclusions can be obtained: (1) the sensing data obtained by the visual e-nose system reflect the information of the input spectrum well; (2) the visual e-nose system can not only detect a single gas, but also has a good detection result for mixed mixture; (3) the classification accuracy of EDC is higher than that of CC, which shows that it is better for EDC algorithm to classify the test data; (4) the average classification accuracy of different algorithms reaches 77%, which verified the effectiveness of the system.

## 5. Conclusions

In this paper, an innovative visual e-nose system based on SHS was proposed. The core of the article was to introduce a WS-SHS into the e-nose system taking its core gas sensing tasks. Additionally, according to the characteristics of the response map derived by the visual e-nose, mature image processing technologies (such as LBP, GLCM) were applied to the data processing of the e-nose to provide a new idea for the development of the e-nose. In addition, the visual e-nose system has the following advantages: Firstly, it has a larger sensor array, faster response time and a wider sensing range which can improve the accuracy and application range of e-nose effectively. Secondly, the use of an optical system can overcome the limitations of the traditional e-nose on high temperature, humidity, corrosivity and provide a useful reference for the research of e-noses. Thirdly, it has good classification results for single gas and mixed gas, indicating that the system has high universality. However, there are still some shortcomings in the article that need to be studied further. On the one hand, there is a need to build a real wide-spectrum visual e-nose prototype; on the other hand, the use of the e-nose prototype to detect a wider range of single or mixed gases should be explored.

## Figures and Tables

**Table 1:** Technical parameters of light absorption gas detection system.

Category	Parameters
Technical principle	Light absorption sensing technology
Response range	200–1100 nm
Size of sensing unit	0.54 nm
Lower limit of detection	NO_2_: 0.2‰; SO_2_: 0.1‰; C_6_H_6_: 0.2‰
Sensitivity	NO_2_: 0.2‰; SO_2_: 0.1‰; C_6_H_6_: 0.1‰
Test gases	Inorganic gas: NO, NH_3_, O_3_, SO_2_, CS_2_, NO_2_, O_2_, etc.
	Organic gas: C_6_H_6_, C_7_H_8_, C_8_H_10_, CH_2_O etc.

**Table 2:** Objective evaluation parameters of sensing data.

Class	Correlation Coefficient				
	NO_2_	SO_2_	NO_2_ + SO_2_	C_6_H_6_	C_7_H_8_
NO_2_	1	0.55	0.67	0.95	0.92
SO_2_	0.55	1	0.44	0.53	0.50
NO_2_ + SO_2_	0.67	0.44	1	0.61	0.58
C_6_H_6_	0.95	0.53	0.61	1	0.94
C_7_H_8_	0.92	0.50	0.58	0.94	1

**Table 3:** Dataset consisting of 2D T-maps.

Gas	NO_2_			SO_2_			NO_2_ + SO_2_			C_6_H_6_	C_7_H_8_
Concentration (‰)	0.2	0.4	0.6	0.3	0.6	0.9	3.2 + 0.1	3.5 + 0.3	3.0 + 0.6	3	3
Number	10	10	12	10	10	12	10	10	12	32	32
Total	32			32			32			32	32

**Table 4:** Classification accuracy of Dataset.

Class	Classification Accuracy (%)			
	CC		EDC	
	LBP	GLCM	LBP	GLCM
NO_2_	100	100	100	100
SO_2_	50	70	80	80
NO_2_ + SO_2_	90	30	100	60
C_6_H_6_	60	70	90	90
C_7_H_8_	60	50	80	80
Mean	72	64	90	82

CC: Correlation coefficient; EDC: Euclidean distance to centroids.
